# *In-Situ* ESEM and EELS Observation of Water Uptake and Ice Formation in Multilayer Graphene Oxide

**DOI:** 10.1038/srep11807

**Published:** 2015-07-02

**Authors:** Takeshi Daio, Thomas Bayer, Tatsuya Ikuta, Takashi Nishiyama, Koji Takahashi, Yasuyuki Takata, Kazunari Sasaki, Stephen Matthew Lyth

**Affiliations:** 1 International Research Center for Hydrogen Energy; 2 Department of Mechanical Engineering; 3 Next-Generation Fuel Cell Center (NEXT-FC); 4 International Institute for Carbon-Neutral Energy Research (WPI-I2CNER); 5 Department of Aeronautics and Astronautics Kyushu University, Motooka 744, Fukuoka, Japan; 6 Department of Mechanical Engineering, University of Sheffield, Sheffield, S10 2TN, United Kingdom

## Abstract

Graphene oxide (GO) is hydrophilic and swells significantly when in contact with water. Here, we investigate the change in thickness of multilayer graphene oxide membranes due to intercalation of water, via humidity-controlled observation in an environmental scanning electron microscope (ESEM). The thickness increases reproducibly with increasing relative humidity. Electron energy loss spectroscopy (EELS) reveals the existence of water ice under cryogenic conditions, even in high vacuum environment. Additionally, we demonstrate that freezing then thawing water trapped in the multilayer graphene oxide membrane leads to the opening up of micron-scale inter-lamellar voids due to the expansion of ice crystals.

Graphene oxide (GO) is receiving increasing attention due to its dispersibility in water^1^, electronically insulating properties^2^, thermal conductivity^3^, catalytic^4^,^5^, gas barrier^6^, molecular sieve^7^, and optical properties^8^. It is being produced at increasingly large scale and the price is decreasing accordingly. GO can be readily dispersed in water, making it very easy to process into single layer, few-layer, or multilayer films by e.g. screen printing, inkjet printing, spin-coating, spraying, or dip-coating. Strong and flexible free-standing GO paper can be manufactured in a similar fashion by bar coating, or vacuum filtration, in much the same fashion as conventional cellulose fiber-based paper^9^. GO is generally obtained by chemical functionalization and exfoliation of graphite via reaction with concentrated sulfuric acid and potassium permanganate using the Hummers method, or variations thereupon. This process introduces oxygen-containing functionality onto the surface, including carboxylic, hydroxyl, epoxy and carbonyl groups^10^. These are responsible for the hygroscopic nature of GO. Such surface functional groups can also act as the basis for further chemical functionalization^11^.

In particular, the carboxylic acid groups also contribute to proton conductivity in graphene oxide^12^,^13^,^14^,^15^, This means that GO can be utilized as an ionomer membrane in proton exchange membrane fuel cells. In our previous work we have shown that the proton conductivity of GO is strongly dependent on relative humidity^16^. GO also has potential applications in water purification and humidity sensors^17^,^18^,^19^,^20^. Recently, low-friction flow of a monolayer of water through the two-dimensional capillaries formed by graphene oxide sheets has been reported^7^. Therefore, studying water uptake and intercalation in GO is of great interest.

Despite the wealth of recent papers published on graphene oxide, *graphite* oxide has been studied in great detail over a much longer timescale^21^,^22^, with applications as a dielectric material or as a lubricant. It is interesting to compare the two similar but subtly different materials. The hydration of graphite oxide has been investigated in several key studies^23^,^24^,^25^. It was established that hydrogen bonding with the surface species drives intercalation of water molecules between the graphite oxide sheets, and this results in expansion of the interlayer spacing along the c-axis, observed macroscopically as swelling^24^. Graphene oxide shows distinctly different hydration properties from its parent graphite oxide, especially in terms on alcohol uptake and the temperature dependence of the interlayer spacing^26^,^27^. In addition, the mechanical properties also strongly depend on water content^28^. Obtaining completely dry graphene oxide (and graphite oxide) has been postulated to be practically impossible, and at least a monolayer of water is likely to be permanently present due to strong hydrogen bonding between water molecules and the surface^18^,^29^.

Direct microscopic observation of the interaction between GO and water has not been as extensively studied. The hydration of bilayer graphene oxide has been investigated by *in-situ* atomic force microscopy^27^, and square ice crystals have recently been observed by electron microscopy in between graphene layers, due to nano-confinement effects^30^. Here, we perform *in-situ* observation of a multilayer graphene oxide cross section over a range of relative humidities, using environmental scanning electron microscopy. Evidence for the existence of water ice intercalated in the multilayer GO membrane is provided by electron energy loss spectroscopy (EELS), even under high vacuum conditions.

## Results and Discussion

### Microscopic observation

**Figure 1:**
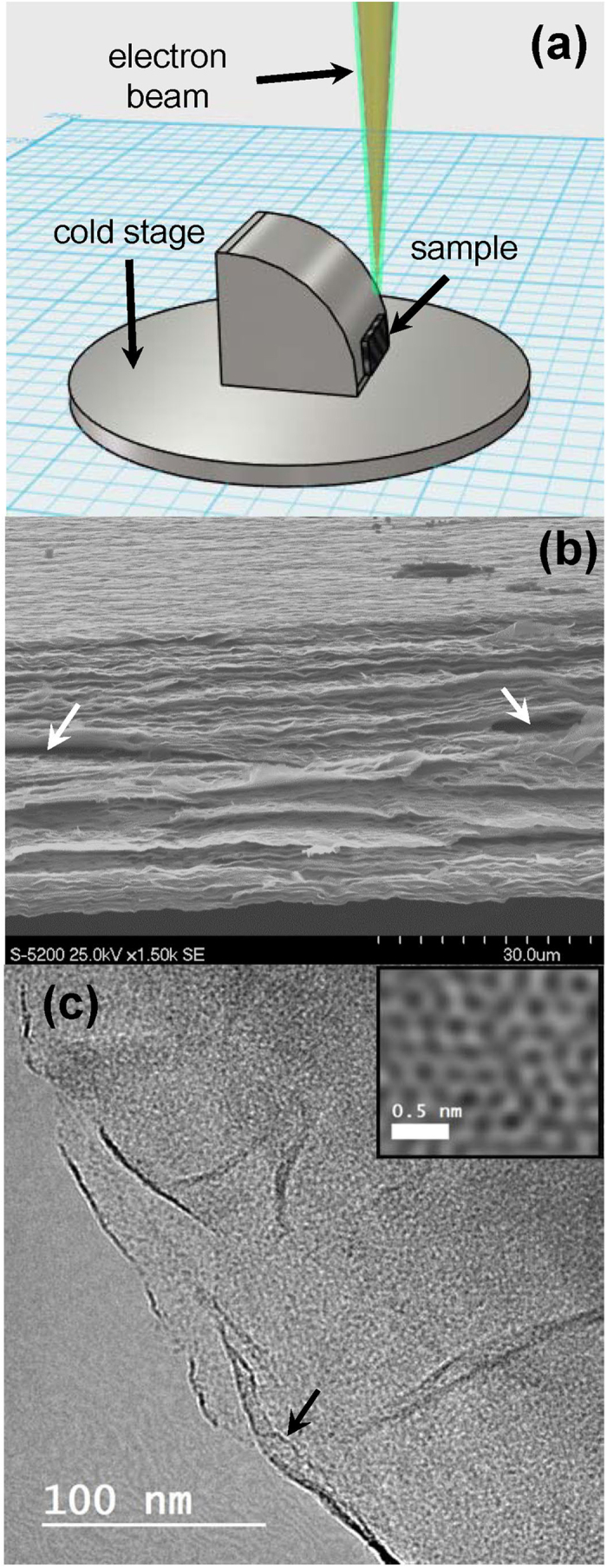
(**a**) Schematic image of the bespoke cold stage. The multilayer GO membrane is attached and the cross-section is exposed to the electron beam. (**b**) Photograph of the stage. (**c**) An SEM image of the multilayer GO membrane. (**d**) TEM images of a few-layer region of the multilayer GO membrane. The inset in (**d**) shows the structure at atomic resolution.

Free-standing multilayer GO paper was produced by vacuum filtration from water dispersion, followed by drying. A bespoke microscope stage was manufactured, allowing side-on observation of the sample (Fig. 1a). Figure 1b shows a typical scanning electron microscopy (SEM) cross-sectional image of the multilayer graphene oxide cross-section. The layered structure can be clearly observed, as well as micron-scale corrugation in the individual GO sheets, and several micron-scale interlamellar voids (white arrows). Figure 1c shows a scanning transmission electron microscopy (STEM) image, taken at a point where few-layer GO protrudes out from the bulk multilayer sample. Two graphene oxide layers can be clearly distinguished. Towards the edge, the upper GO sheet is slightly folded back on itself (black arrow). The inset shows an atomic resolution image. The hexagonal arrangement of carbon atoms can be clearly observed, as well as many vacancies and dangling bonds associated with the harsh oxidization process^31^.

### ESEM analysis

**Figure 2:**
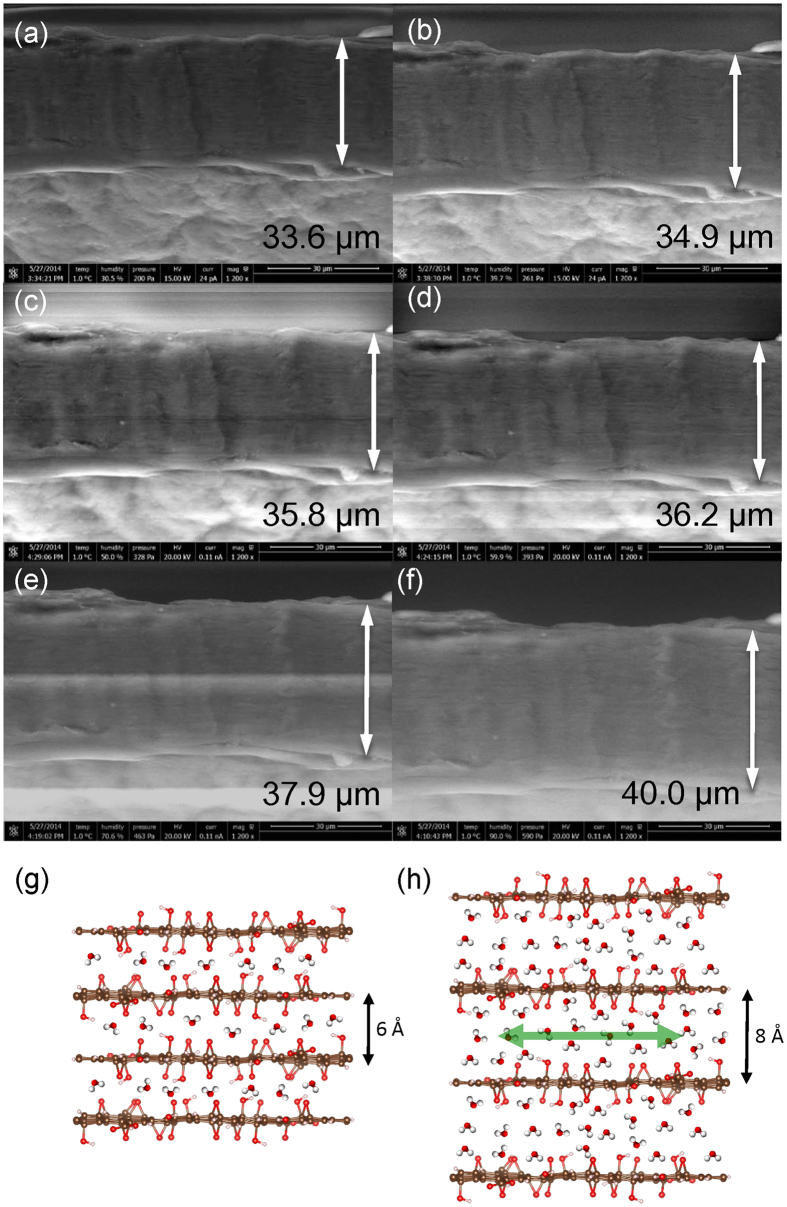
ESEM images of the cross-section of the multilayer GO membrane at (**a**) 30, (**b**) 40, (**c**) 50, (**d**) 60, (**e**) 70, and (**f**) 90 % RH. Schematic images of few-layer GO under (**g**) dry and (**h**) humidified conditions.

The effect of humidity on the membrane was measured by *in-situ* ESEM. The thickness was first measured at a fixed point on the sample, which was used as a reference area at different relative humidity, under fixed focus conditions. The relative humidity was increased stepwise from 30% RH to 95% RH, then decreased following the same data points. This cycle was repeated twice. To ensure saturation between thickness measurements, images were taken 120 seconds after each step; response times of the order 10 to 20 seconds are reported in the literature^19^,^20^. The response in this case is estimated to be of the order of 5 seconds (as shown in the video file in SI). SEM images obtained at different relative humidity are shown in Figure 2. Figs 2g,h show simple schematic representations of the increase in total thickness and interlayer spacing due to intercalation of water molecules. In Fig. 2g, a monolayer of bound and relatively immobile water is assumed to be present under dry conditions. At higher relative humidity confined- and/or bulk-type water are also assumed to be present (Fig. 2h).

**Figure 3:**
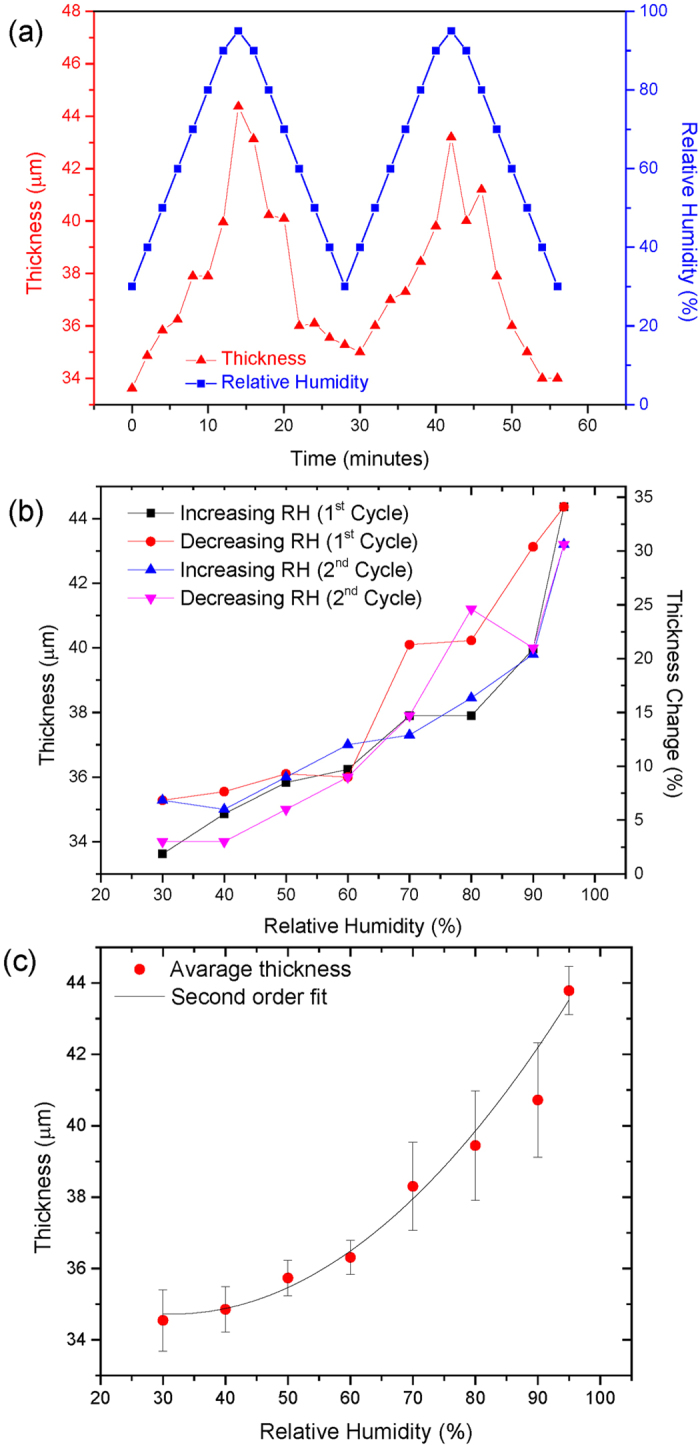
(**a**) Relative humidity and resulting thickness variation of the multilayer GO membrane with time. (**b**) Thickness versus relative humidity. (**c**) Average thickness at a given relative humidity, with second order fitting.

The variation in thickness of the multilayer graphene oxide cross-section with relative humidity is plotted in Fig. 3. Figure 3a shows the change in thickness over time, with two successive cycles in RH. It is evident that the change in thickness closely follows the relative humidity, and the trend is similar to that observed in previous studies, using different techniques^25^,^27^. Figure 3b shows the time-independent dependence of thickness on RH. There does not appear to be significant hysteresis between the up- and down- cycles, reflecting the fast kinetics of the interaction between water and graphene oxide. The thickness change is relatively reproducible between cycles. Figure 3c shows the averaged data with a 2^nd^ order fit (R^2^ = 0.983). The thickness of the multilayer GO membrane increases from 34.5 μm at 30% RH to to 43.5 μm at 95% RH, corresponding a total increase of around 26%. The thickness of bi-layer GO measured by atomic force microscopy has previously been measured to vary from 0.74 nm at 30% RH, to 0.85 nm at 90%RH; an increase of 14.9%^27^. In this work, the change in thickness is 18.0% over the same range of RH, and we previously measured the interlayer spacing to be 0.76 nm at 30% relative humidity^16^. Our results agree quite well with the literature and demonstrate how the nanoscale properties of graphene oxide can translate to scaled-up systems. The small discrepancy between the two values may arise from simple scaling; the outer surface layers of water must be taken into account in bi-layer graphene oxide, but the thickness of these become negligible the multilayer system. Additionally, the discrepancy may arise due to differences in the oxygen content, the types and ratios of functional groups on the surface, or the flake size of the different GO materials used in each study.

Interestingly, although a simple linear dependence of thickness on relative humidity might be expected, the thickness increase is more pronounced at higher relative humidity. This behavior is also seen in other studies on graphite oxide^25^, but has not been discussed in detail. Water intercalated between the layers of hydrated graphite oxide is believed to be present in three different states^23^,^25^. *Bound* water is relatively immobile and is strongly adsorbed to the adjacent GO by hydrogen bonds, forming a surface monolayer. This may be the only state of water present under “dry” conditions. *Confined* water is more mobile and is free to move in two dimensions. *Bulk* water behaves like normal water without constraints and is likely to be present at high relative humidity. The non-linear dependence of the thickness on relative humidity may relate to the differences between these different states of water. Specifically in the case of bound water, the graphene oxide sheets are also expected to be more tightly bound. When bulk-type water is present in the interlayer region the interaction between individual graphene oxide sheets should be much weaker, allowing for more facile intercalation of water molecules under high humidity conditions. Step-wise increases in thickness with relative humidity associated with filling of the interlamellar spaces with discreet monolayers of water were not observed as in some previous studies^32^. The gradual change in thickness and the masking of this step-wise increase in thickness is attributed to the averaging effect of having several thousand stacked monolayers.

**Figure 4:**
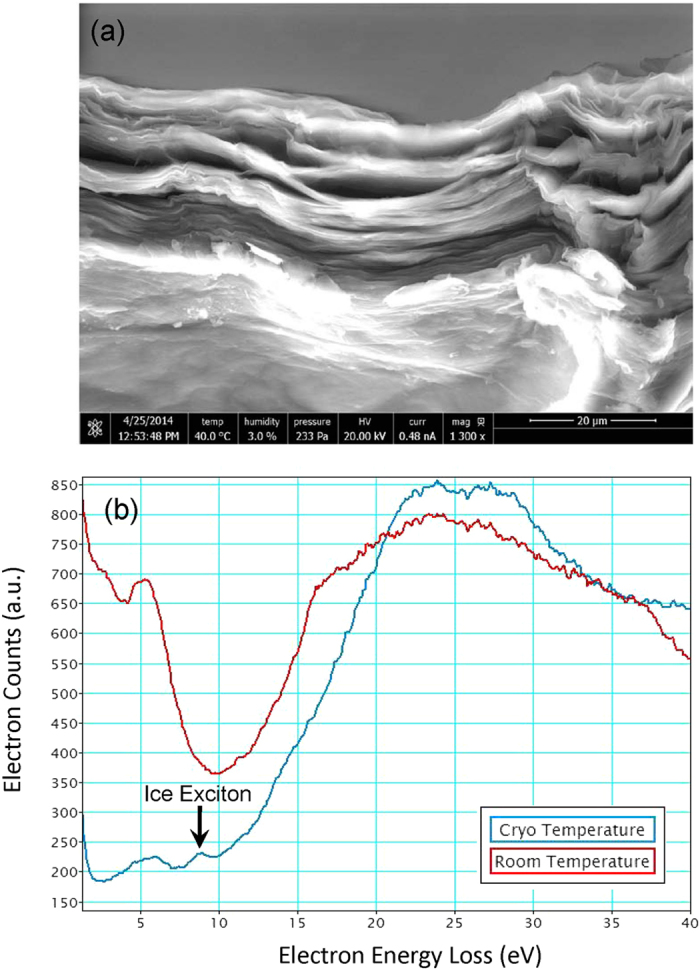
(**a**) SEM image of the multilayer GO membrane after freezing then thawing. (**b**) EELS spectra of GO at 40 °C and −160 °C. The peak at 8.5 eV, signifies the presence of crystalline ice. The peak at 5 eV is characteristic of GO.

The effect of cryogenically freezing the multilayer GO was also investigated by ESEM. The relative humidity was maintained at 80%, and the sample stage was rapidly cooled from 1 °C to −20 °C. Following this, the multilayer GO sample was quickly heated to 40 °C. Upon thawing, extensive changes in the morphology were observed (Fig. 4a). Corrugation of the multilayer graphene oxide membrane is dramatically increased, and multiple micron-scale interlamellar voids are opened up in the cross section. This significant change in morphology may be explained by pockets of water ice forming in larger voids in the membrane (as observed in Fig. 1(b)). Upon thawing, the water ice evaporates or sublimes, increasing the vapor pressure and forcing apart the graphene oxide layers, resulting in severe mechanical deformation.

### EELS Characterization

The low-loss region of EELS spectra is a direct measure of the dielectric response to external electromagnetic excitation. This was recorded at high vacuum, on a few-layer region of the multilayer graphene oxide membrane sample first at 28 °C, and then after cooling to −160 °C (Fig. 4b). The samples were stored at ~60% RH before being placed in the sample chamber. Three main peaks are observed in the room temperature EELS spectrum. The first is the zero-loss peak, caused by elastically scattered electrons. The loss peak at around 5 eV is attributed to the π → π* excitation^33^. The peak at around 24 eV is attributed to the π + σ bulk plasmon loss (π* + σ* excitations)^34^. The shoulder at around 16 eV may be attributed to σ → π* and π → σ* single electron transitions^35^. These results are fairly typical for room temperature EELS characterization of graphene oxide. However, at cryogenic temperature, a new peak appears at around 9 eV. This peak position is consistent with similar EELS studies on water ice^36^,^37^,^38^,^39^, and corresponds to water molecule excitation. This peak is broader and lower in intensity than that expected for free ice, and this may be due to the scattering effect of carbon surrounding the ice, and nano-confinement between the graphene oxide layers. Such nano-confinement has recently been shown to produce square water ice crystals in multilayer graphene^30^. Since the sample was observed under high vacuum for an extended amount of time before cooling, any water contamination on the outside surface of the sample is expected to have sublimed. From these EELS spectra, it is clear that water is intercalated in multilayer graphene oxide even under high vacuum conditions. This confirms that water is extremely strongly bound onto graphene oxide, and that completely “dry” graphene oxide is likely to be extremely difficult to isolate.

## Conclusion

In conclusion, we performed *in-situ* SEM and *in-situ* STEM characterization of multilayer graphene oxide (GO) at different temperatures and relative humidities. The thickness increased gradually with increasing relative humidity, and decreased with decreasing relative humidity. The rate of change in thickness was slower at low relative humidity, suggesting stronger interlayer bonding in drier conditions. Freezing and then thawing the membrane results in strong corrugation of the multilayer graphene oxide membrane as well as the introduction of micron-scale void throughout the cross section. EELS analysis of few-layer graphene oxide under cryogenic conditions revealed that water ice is present even after exposure to high vacuum, suggesting that obtaining completely “dry” graphene oxide is extremely challenging. Intercalation of water inside graphene oxide could be a significant advantage in applications such as fuel cell ionomer membranes, where water uptake even at high temperature is required.

## Methods

### Membrane Preparation

Multilayer graphene oxide was prepared by vacuum-filtration of GO dispersion (Graphene Supermarket, Highly Concentrated Graphene Oxide Dispersion in Water, 5 g/L; Composition: Carbon (79%), Oxygen (20%); Flake size: 0.5–5 μm; Thickness: 1 atomic layer - at least 60%; Hummers Method) onto PTFE Millipore filters (diameter 35 mm, pore size 0.025 μm). The retentate was dried at room temperature for 48 hours and then peeled-off the filter, resulting in a freestanding multilayer grapehen oxide paper-like membrane. The cross section for SEM investigation was prepared by shearing a thin strip GO paper using a tensile strength test machine (SHIMPO FGO-C-TV), resulting in a clean fracture. Simply cutting the membrane with a scalpel generally does not result in a clean cross section, due to extensive slipping of the GO sheets.

### ESEM Analysis and Characterization

SEM images were acquired using a dual-beam focused ion beam system (Versa, FEI), equipped with a cold stage and a humidifier. The acceleration voltage was 20 kV. Samples were mounted on sticky-coated copper tape (Okenshoji, Japan) which was then attached to a bespoke sample stage, allowing the sample edge to be tilted towards the electron gun in order to be able to observe the cross-section. A schematic image of the set-up is shown in Fig. 1. To achieve control over the relative humidity in the low pressure environment, the stage was generally maintained at 4 °C, unless otherwise stated.

### TEM Analysis and Characterization

STEM images were acquired using a Cs-corrected STEM (JEOL, JEM-ARM200F), equipped with a liquid nitrogen cryo-holder (JEOL). The acceleration voltage was limited to 80 kV to avoid knock-on damage of the GO specimen. A spectrometer (Enfinium, Gatan, US) attached to the microscope was used for EELS analysis. The energy resolution is 0.5 eV at the full-width half-maximum (FWHM) of the elastic peak. The samples were mounted on double folding grids (Ted Pella, US). The *in-situ* EELS experiment was performed after 1 hour at room temperature and 1 × 10^−5^ Pa, then after 1 hour at −160 °C.

## Additional Information

**How to cite this article**: Daio, T. *et al.* *In-Situ* ESEM and EELS Observation of Water Uptake and Ice Formation in Multilayer Graphene Oxide. *Sci. Rep.* **5**, 11807; doi: 10.1038/srep11807 (2015).
